# Tunnel junction based memristors as artificial synapses

**DOI:** 10.3389/fnins.2015.00241

**Published:** 2015-07-07

**Authors:** Andy Thomas, Stefan Niehörster, Savio Fabretti, Norman Shepheard, Olga Kuschel, Karsten Küpper, Joachim Wollschläger, Patryk Krzysteczko, Elisabetta Chicca

**Affiliations:** ^1^Thin Films and Physics of Nanostructures, Bielefeld UniversityBielefeld, Germany; ^2^IFW Dresden, Institute for Metallic MaterialsDresden, Germany; ^3^Cognitive Interaction Technology Center of Excellence and Faculty of Technology, Bielefeld UniversityBielefeld, Germany; ^4^Fachbereich Physik and Center of Physics and Chemistry of New Materials, Osnabrück UniversityOsnabrück, Germany; ^5^Physikalisch Technische BundesanstaltBraunschweig, Germany

**Keywords:** memristors, artificial synapses, tunnel junction, synaptic plasticity, neuromorphic systems

## Abstract

We prepared magnesia, tantalum oxide, and barium titanate based tunnel junction structures and investigated their memristive properties. The low amplitudes of the resistance change in these types of junctions are the major obstacle for their use. Here, we increased the amplitude of the resistance change from 10% up to 100%. Utilizing the memristive properties, we looked into the use of the junction structures as artificial synapses. We observed analogs of long-term potentiation, long-term depression and spike-time dependent plasticity in these simple two terminal devices. Finally, we suggest a possible pathway of these devices toward their integration in neuromorphic systems for storing analog synaptic weights and supporting the implementation of biologically plausible learning mechanisms.

## 1. Introduction

Memristors have attracted great interest for a variety of applications in recent years (Prezioso et al., 2015). An obvious use would be as a memory device (Chen et al., 2008; Linn et al., 2010; Lee et al., 2011) or, more ambitiously, a reconfigurable logic device (Borghetti et al., 2009; Xia et al., 2009; Borghetti et al., 2010; Muenchenberger et al., 2011; Yan et al., 2011). However, the arguably most interesting implementation of memristive devices is neuromorphic computing (Jo et al., 2010; Indiveri et al., 2013).

Neuromorphic engineering is a relatively young research field, which was originally proposed by Mead (1989, 1990) in the late 80s. Neuromorphic devices and architectures are designed to emulate the style of computation of biological systems and exploit biological strategies for optimizing robustness to noise and fault tolerance, as well as maximizing compactness and minimizing power consumption. Nevertheless, the most attractive feature of biological systems is their ability to learn and adapt to new situations. Artificial agents equipped with such abilities would have a broad range of applications. The possibility of embedding learning capabilities in neuromorphic systems is therefore extremely appealing. One route toward implementing these synaptic weights is the memristor. However, well-characterized materials suitable for the construction of memristive devices with large memristive switching are needed. In this manuscript we investigate possible solutions to this problem.

A possible realization of a memristive device is a metal-insulator-metal structure. In particular, this can be a tunnel junction. Then, a 1–3 nm thin insulator separates two metal electrodes, and the tunneling current is determined while the bias voltage is applied. The scalability of (magnetic) tunnel junctions was already shown in magnetic random access memory devices, and a 16 Mb chip is commercially available. Consequently, we suggest the use of memristive tunnel junctions as artificial synapses in neuromorphic circuits.

**Figure 1:**
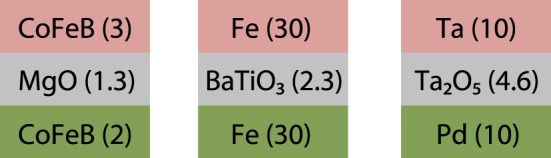
**Typical functional layer stacks of the presented memristive tunnel junctions**. The thickness of the individual layers are given in parentheses in nm.

In this manuscript, we will present several oxides used as the barrier materials in tunnel junctions. A cartoon of the layer stacks is depicted in Figure 1. Many oxides can exhibit memristive switching behavior in such a tunneling structure, which allows us to tailor the electrode and barrier materials for a given application. First, we introduce magnesia based tunnel junctions and use them to look into the analogs of long-term potentiation, long-term depression, and spike-time dependent plasticity, which are the basic functional properties of biological synapses (Thomas, 2013). However, the resistance change in these junctions is rather small [(*R*_max_ − *R*_min_)/*R*_min_ = 8%]. Consequently, new material combinations are tested, and we will show resistive switching in BaTiO_3_ and Ta-O based systems. BaTiO_3_ and TaO based junctions exhibit up to 10 times larger resistance changes, which improves the prospects of these systems in semiconductor-based neuromorphic circuits. The general suitability of these junctions is discussed in the last section and compared to the requirements suggested by Indiveri et al. (2013).

## 2. Materials and methods

### 2.1. Magnesia based tunnel junctions

The MgO-based magnetic tunnel junctions are sputtered on SiO_2_ generated by thermal oxidation of Si. The bottom layer stack consists of Ta/Cu−N/Ta/Pt−Mn/Co−Fe/Ru, the functional tunnel system is composed of Co−Fe−B/MgO/Co−Fe−B, while Ta/Cu/Ru form the top layers. The deposition is performed at room temperature and followed by a post-annealing step at 360°C for 90 min. Afterwards, elliptical tunnel junctions with a major (minor) axis of 350 nm (150 nm) are prepared by successive steps of electron beam lithography and ion beam etching. More details on the preparation and characterization of magnesia based tunnel junctions are given in previous publications (Krzysteczko et al., 2008, 2009).

All measurements were carried out at room temperature with a voltage source. The voltage pulses of up to 800 mV have 1 s duration and lead to a current density of 1 × 10^6^ A/cm^2^ to 10 × 10^6^ A/cm^2^. These values are close to the dielectric breakdown voltage of the devices. Transmission electron microscopy images of MgO junctions before and after the dielectric breakdown are presented in our previous work (Thomas et al., 2008; Schaefers et al., 2009). Consequently, the investigations utilizing sequences of voltage pulses were limited to a maximum voltage of 500 mV. The resistance of the device is determined 200 ms after the write pulse by measuring the current at a voltage of 20 mV. The base resistance of the layer stack is approximately 35 Ωμ*m*^2^ leading to a resistance of approximately 200 Ω for the given lateral junction size. The current flows from the top to the bottom electrode at positive bias voltages.

### 2.2. Barium titanate junctions

The preparation of barium titanate (BTO) is more challenging than the other materials, because its tetragonal phase with perpendicular orientation of the *c*-axis is required. Therefore, suitable substrate and electrode materials are essential. The lattice constant of MgO is 4.21 Å (Rocksalt structure), while that of iron is 2.86 Å (bcc). Because of the cubic lattice structure of iron and magnesia, the lattice mismatch is 4% with respect to two unit cells of Fe in the diagonal direction to one unit cell of MgO [Fe(001);<110>||MgO(001);<100>]. Additionally, the lattice mismatch between Fe and BTO (001) is 1.4% in the diagonal growing case, making this system a good candidate for coherent tunneling junctions. Therefore, we chose Fe/BTO/Fe tunneling junctions prepared by rf-magnetron sputtering.

Because of both the small lattice mismatch between Fe and MgO and reliable tunneling junctions of Fe/BTO/Fe, we chose MgO (100) substrates. BTO films were prepared by rf-magnetron sputtering from a single BTO-target (3 inch) with an applied power of 50 W and an argon pressure of 2.1 × 10^−3^ mbar. The substrate temperatures *T*_*S*_ during the BTO deposition was varied from room temperature up to 918°C. The crystal structure was investigated by X-ray diffraction (XRD) using Cu-K_α_ radiation, and the film thickness was measured by X-ray reflectrometry (XRR). After deposition the composition was measured by XPS (Ba 21%, Ti 21%, O 58%) for *T*_*S*_ = 737°C and is close to the stoichiometric ratio of 1:1:3. The tunneling junctions were structured with standard optical lithography techniques. The junction area of the samples presented in this manuscript is 25 μm × 25 μm. The junction resistance was approximately 4 kΩ at an applied voltage of 10 mV.

Initially, we characterized our BTO films directly sputtered on MgO by XRD. The film thickness was determined by XRR to calibrate the sputtering. Then, the thickness was set to 10 nm for all samples.

**Figure 2:**
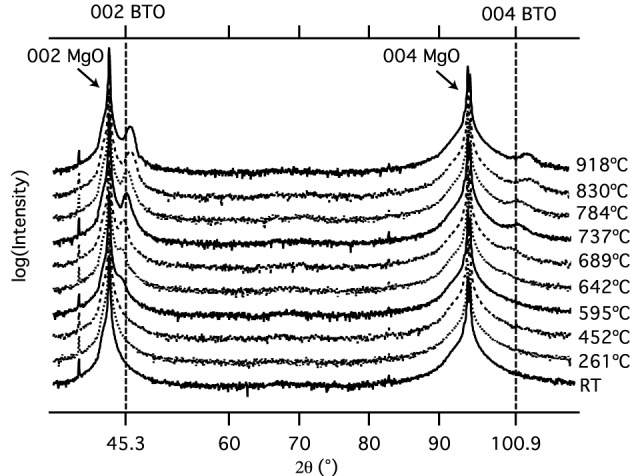
**X-ray diffraction pattern of the barium titanate films at different deposition temperatures on MgO substrates**. An increase of the 002 and 004 BTO-peaks is observed with increasing substrate temperature.

Figure 2 shows the XRD patterns of the BTO films deposited at the given temperature *T*_*S*_. Both the BTO (002) and the BTO (004) peak intensities increase with increasing temperature up to 918°C. Furthermore, the position of the BTO (002) peak shifts from 44.425° at *T*_*S*_ = 689°C to 45.925°C at *T*_*S*_ = 918°C, corresponding to a decrease in the *c*-axis lattice constant from 4.078 Å down to 3.954 Å, which is shown in detail in Table 1.

**Table 1:** **The** ***c*****-axis lattice parameters of BTO (10 nm) layers on MgO substrates calculated via the 002 peak at different deposition temperatures**.

*T* [°C]	595	642	689	737	786	830	918
*c* [Å]	4.078	4.062	4.035	3.997	3.989	3.951	3.954

*At RT, 261 and 452° no peak is visible in the spectra*.

The *c*-axis lattice parameter of the sample at T_*S*_ = 689°C exhibits a lattice constant of *c* = 4.035 Å, which closely agrees with the c-axis lattice parameter of *c* = 4.036 Å (Kim et al., 1995). Four phases are possible in BTO: rhombohedric, orthorhombic, tetragonal, and cubic. We can assume that we have achieved a tetragonal phase with perpendicular orientation of the *c*-axis, which is in good agreement with results previously reported by Kim et al., who fabricated epitaxial BTO films onto MgO (001) substrates (Kim et al., 1995).

### 2.3. Tantalum oxide

All films are fabricated by dc magnetron sputter deposition with a base pressure of 3.5 × 10^−7^ mbar and a sputter pressure of 1.3 × 10^−3^ mbar. The junctions are defined by optical lithography and argon ion beam etching leading to a size of 10 × 10 μm.

We used Pd as the bottom and Ta as the top electrode to generate asymmetric barrier interfaces, both electrodes with a thickness of 10 nm. Our 4.6 nm Ta-O film was produced by plasma oxidation of a 2 nm Ta film as previously reported by Park and Im (1992). The penetration depth is regulated by the bias voltage of the plasma (Rottländer et al., 2001; Thomas et al., 2003). The oxygen concentration was regulated by the oxidation time to generate a tunneling barrier with a high concentration of oxygen vacancies. The generation and movement of oxygen vacancies at one interface in an electrical field results in the resistance change (Krzysteczko et al., 2009; Yang et al., 2010).

## 3. Results and discussion

### 3.1. Magnesia based tunnel junctions

**Figure 3:**
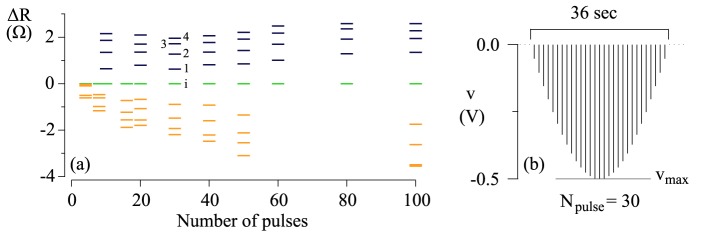
**(A)** Long-term potentiation and long-term depression of an MgO-based magnetic tunnel junction. The refreshed state is set to zero (green bars). The relative resistance increase is shown in blue and the relative resistance decrease in orange. **(B)** A cartoon of an example pulse sequence of 30 pulses. It consists of a series of 1 s rectangular pulses, convoluted by a sinusoidal half-wave with the amplitude *v*_max_. The pulses are separated by 200 ms intervals. For similar pulse sequences, the pulse widths and pulse intervals are always fixed (1 s and 0.2 s, respectively) and the sine function involved in the convolution has a half-period given by the total duration of pulse sequence.

The resistance change of the MgO junctions is determined by a number of voltage pulses, leading to a relative change in resistance as depicted in Figure 3A. First, we will look into one example, where a pulse sequence of 30 voltage pulses is used, which corresponds to the bars of Figure 3A marked with digits i and 1–4. The pulse sequence itself is described by the cartoon in Figure 3B and is inspired by biological data (Rose and Dunwiddie, 1986). The initial state is indicated by the green bar marked with an i.

Now, one pulse sequence (30 voltage pulses) of the described shape is applied to the tunnel junction, and the resistance increases to the value indicated by the digit 1. An additional pulse sequence further increases the resistance to the value signified by the digit 2. Subsequently, the resistance was increased by several pulse sequences finally leading to the value indicated by the digit 4. We observe a systematic decrease of the resistance change after every pulse, and eventually the resistance saturates and reaches its maximum or minimum value. This is true for all memristive devices (Chua, 2014).

This was also performed for pulse sequences with a different number of voltage pulses. The number of voltage pulses in each pulse sequence is given by the abscissa in Figure 3A. We carried out these measurements for negative voltages as depicted in Figure 3B, leading to a resistance increase, which corresponds to the blue bars in Figure 3A. A resistance decrease is caused by positive voltages, which corresponds to the orange bars in Figure 3A. The maximum voltage was kept constant at |*v*_max_| = 500 mV in all cases.

**Figure 4:**
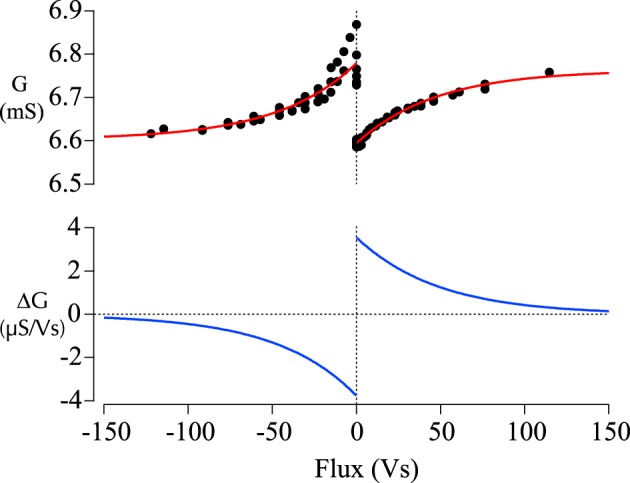
**Flux-dependent plasticity of memristive magnetic tunnel junctions. Top:** The asymmetric conductivity of a memristive tunnel junction. Positive flux is associated with causal spike-timing, negative flux with anti-causal spike-timing. The asymmetry typical for stdp is evaluated by the fitting curves. **Bottom**: The derivative of the fitting curves in the top graph is calculated to provide a measure of the change in synaptic strength.

The increase (decrease) in the conductivity of the junctions can be associated with long-term potentiation LTP (depression, LTD) in a biological neural network. Linares-Barranco et al. suggested to shape the pulses in a particular way (see Linares-Barranco and Serrano-Gotarredona, 2009): The increasing and decreasing edges of the pulses follow an exponential increase and decay, respectively. Two subsequent pulses can generate a positive as well as negative net flux. If we now take similar data depending on the spike-timing, we acquire the flux-dependent plasticity shown in Figure 4.

If we fit the collected data, we obtain

$$G(\varphi )=\frac{1}{148\text{ mS}+3.77\text{mS}\cdot \exp(-\varphi /46.3\text{Vs})}$$

for positive as well as

$$G(\varphi )=6.6\;\times \;10^{-3}\text{mS}+0.177\;\times \;10^{-3}\text{mS}\cdot \exp(\varphi /47\text{Vs})$$

for negative values, where *G* denotes the conductance and φ denotes the flux. This leads to the red fitting curves in Figure 4 and can be used to calculate the relative change in conductance also depicted in Figure 4 in blue. The same kind of functions can be used to fit biological data, e.g., by Bi et al. (Bi and Poo, 1998). Therefore, we are able to show behavior similar to spike-timing dependent plasticity (STDP) in these simple two terminal devices (Jo et al., 2010; Krzysteczko et al., 2012). In the following sections, we suggest more barrier materials to be used in memristive tunnel junctions. We aim to find larger resistance changes than up to 8% in magnesia based tunnel junctions. Furthermore, we would like to show that memristive switching is frequently observed in these kinds of structures.

### 3.2. Barium titanate junctions

Initial success with memristive BTO tunnel barriers was published by Chanthbouala et al. on LSMO/BTO/Co systems, in which junction preparation was accomplished by pulsed laser deposition (Chanthbouala et al., 2012). Therefore, we looked into sputtered memristive tunnel junctions based on BTO barriers (Hippel, 1950; Kim et al., 1995). In other contexts, this compound is best known for its ferroelectric properties, which are caused by the tetragonal crystal structure. In the future, this might lead to multi-functional tunnel junctions (Fiebig, 2005; Tsymbal, 2006), i.e., junctions exhibiting memristive behavior and ferroelectricity at the same time.

**Figure 5:**
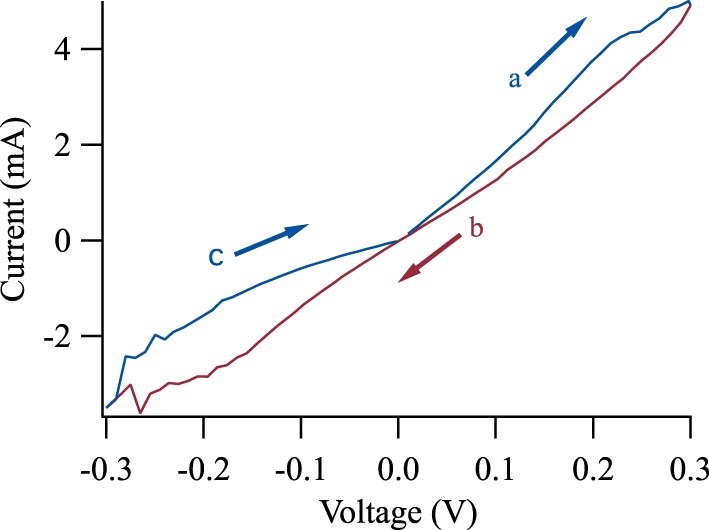
**Current-voltage characteristics of a BTO based tunnel junction**. The measurement sequence is a, b, c at an applied voltage range of −300 to 300 mV. The hysteresis loop shows a type II non-crossing hysteresis.

Figure 5 shows a tunneling hysteresis loop in the applied dc bias range of −300 to 300 mV for *T*_*S*_ = 737°C. The measured sequence was from 0 mV up to 300 mV, down to −300 mV and back to 0 mV. The time between each data point was 200 ms. The figure displays the pinched hysteresis loop characteristic for all memristors (Chua, 2014). According to the theoretical overview of Pershin et al., memristors can be categorized into two types (Pershin and Di Ventra, 2011): Self crossing and non-self-crossing, i.e., the two branches of the hysteresis loop do or do not cross each other.

For BTO, we are interested in the unusual non-crossing hysteresis loop, since most memristive systems show a crossing, i.e., a type-I behavior. A non-self-crossing hysteresis loop can be observed in thermistors and elastic memcapacitive systems. In contrast, Chanthbouala et al. observed a crossing (type-I) I-V-curve for a Lsuppa-Sr-Mn-O/BTO/Co system (Chanthbouala et al., 2012).

However, the memristive effect is very sensitive to the preparation of the junctions, as observed and discussed by Krzysteczko et al. for MgO-based systems (Krzysteczko et al., 2008, 2012). Our BTO system consists of Fe electrodes and is prepared by magnetron sputtering. Chanthbouala et al. grew their junctions using pulsed laser deposition and discussed the influence of structure on the nucleation and propagation of domain walls. The Kolmogorov-Avrami-Ishibashi model describes clean (epitaxial) systems, in which switching is predominantly caused by domain wall propagation (Ishibashi and Takagi, 1971; Hashimoto et al., 1994; Jo et al., 2009). In disordered systems, nucleation-limited switching models should be used (Du and Chen, 1998; Tagantsev et al., 2002), which might explain the different crossing behaviors, even for similar systems utilizing BTO tunnel barriers. The combination of crossing as well as non-crossing behavior might be exploited for compact sequential logics as suggested by You et al. (2014).

BTO-based memristive systems are promising because of their large amplitude of resistive switching. Figure 5 exhibits an 80% change in the tunneling current at −200 mV, which is approximately 10 times larger than the amplitude in our previously investigated MgO-based tunnel junctions (Krzysteczko et al., 2008, 2012). Furthermore, the epitaxial BTO systems indicate the potential of the BTO tunneling systems that exhibit resistance changes of a factor of 750 for 3 nm films (Garcia et al., 2009). However, the epitaxial growth might pose additional challenges for the integration in existing neuromorphic circuits.

### 3.3. Tantalum oxide

Previous reports of Ta-O-based memristive devices show a fast and stable switching behavior for at least 1 × 10^10^ cycles (Yang et al., 2010; Torrezan et al., 2011). However, the thickness of the Ta-O layer always exceeded 7 nm. Consequently, we prepared tunnel junction type systems with a Ta-O barrier.

**Figure 6:**
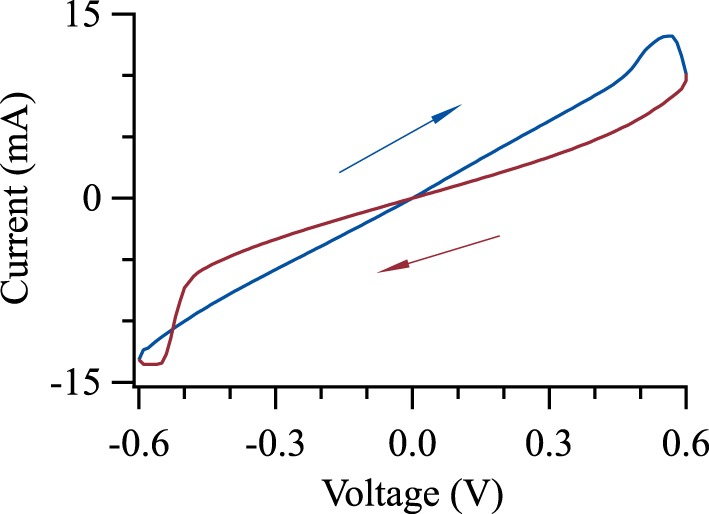
**Memristive switching of Pd-TaO-Ta tunnel junction**. The barrier was oxidized for 150 s leading to the largest ratio between the high and low resistance states.

The samples with oxidation times of 150 s and 200 s reached the highest ratio between the lowest and highest resistance states. Figure 6 shows an *I-V*-loop of a junction oxidized for 150 s, we observed values of up to 80% in the depicted junction. We swept the voltage from zero to −600 to 600 mV and back to zero. Voltages of more than 600 mV led to a dielectric breakdown of the junctions (Thomas et al., 2008; Schaefers et al., 2009). All measurements are done with the bottom electrode as the reference potential.

A Brinkman-Fit (Brinkman et al., 1970) of our measurements shows a Ta-O barrier with an effective thickness of 1.7–1.9 nm, a height in the range of 0.90–1.12 eV and an asymmetry in the range of 0.08–0.35 eV. The difference between the effective barrier thickness and the measured thickness of the Ta-O film seems to result from the post-sputtering *in-situ* oxidation, which generates a rough interface to the bottom electrode.

**Figure 7:**
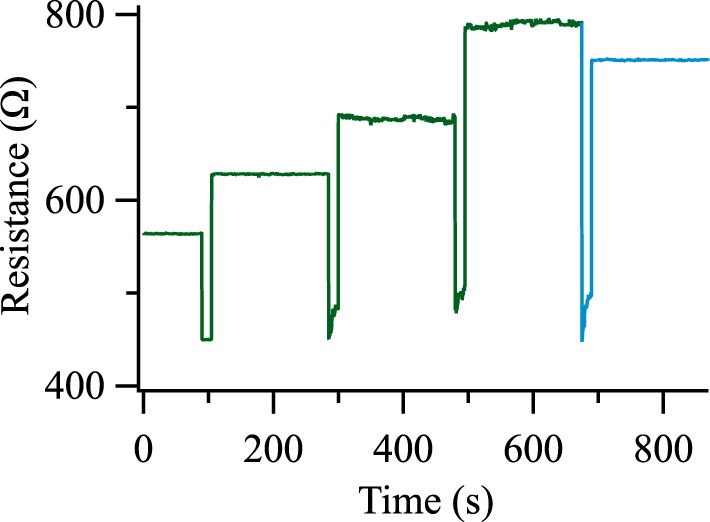
**Analogs of long-term potentiation and long-term depression of a Ta-O based tunnel junction**. Positive voltage pulses are depicted in green, negative voltage pulses in blue. The pulse voltage was ±600 mV in all cases.

Furthermore, we are able to reach more than two states in a Ta-O based tunnel junctions, as shown in Figure 7. We generate the resistance steps by applying a voltage of ±600 mV for 15 s. The resulting resistance levels are measured with a voltage of 10 mV for 180 s. The first three positive (green) voltage pulses increase the resistance while the last (blue) negative pulse decreases it. We can observe the analog of long-term depression and long-term potentiation in Ta-O based junctions, and we have increased the signal by more than a factor of 10 compared to 10% resistance change in MgO based systems. This could emulate the synaptic weight in a neuromorphic chip, and a possible future implementation is suggested in the fifth section.

## 4. Alternate mechanisms for memristive/resistive switching

There are several other mechanisms that can lead to memristive or resistive switching in mesoscopic systems (Waser et al., 2009): Nanomechanical effects, molecular switching, electrostatic/ electronic effects, electrochemical metallization, valence change, thermochemical effects, phase change, magnetoresistance, ferroelectricity, and the presented change in the effective tunnel barrier thickness. In the next paragraphs, we will present a few published results based on different mechanisms. However, this is not an extensive review of the current state of the art, but rather a comparison of several differences and similarities to emphasize the current challenge for memristive tunnel junctions: The amplitude of the resistance change.

Redox-related chemical effects—which include electrochemical metallization, valence change, and thermochemical effects—were intensively studied (Waser and Aono, 2007), even before the term *memristor* moved back into the focus of attention (Strukov et al., 2008). A lot of the research interest was driven by the search for a non-volatile memory that could replace Flash at some point in the future (Waser, 2008).

We start with systems based on electrochemical metallization. These devices consist of a trilayer of an electrochemically inert material, an electrochemically active material and a thin film electrolyte sandwiched in between the two (Kozicki and Mitkova, 2008). Sometimes, this mechanism is also called conductive bridging or programmable metallization cell. An applied voltage leads to a formation or dissolution of a metal filament between the two electrodes, decreasing or increasing the resistance of the device, respectively. The direction of the process depends on the polarity of the applied voltage, which is consequently denoted as bi-polar switching. An *R*_off_/*R*_on_ ratio [resistance in the on (*R*_on_) and off (*R*_off_) states] of more than 1 × 10^5^ and switching times of less than 100 ns were reported (Waser et al., 2009). However, often a forming step of voltages on the order of 5 V is required before the switching voltage of approximately 1 V can be used.

The change of the resistance in a device can also be induced by thermal effects. These mechanisms do not depend on the direction of the current flow. Therefore, these mechanisms always have unipolar characteristics. Two prominent examples are thermochemical switching and phase change materials (Kuzum et al., 2012). The first case can be observed in, e.g., transition metal oxides. Gibbons and Beadle investigated this already in the 1960s in Ni-O (Gibbons and Beadle, 1964). The on state is also caused by a filament connecting the two electrodes (Waser et al., 2009) and an on/off ratio of two orders of magnitude is reported, e.g., for Pt/Ni-O/Pt devices (Yun et al., 2007). In the second case, a structural phase change causes the resistance change. Simpson et al. report, e.g., on GeTe/Sb2Te_3_ devices (Simpson et al., 2011). The resistance change of two orders of magnitudes was induced by voltage pulses of several volts for 50–100 ns. Similar resistance changes were reported by Eryilmaz et al., although the amplitude of this change decreases to 100% if a continuously varying resistance is desired (Eryilmaz et al., 2014).

The research investigating magnetic as well as ferroelectric systems was looking into their use as magnetic or ferroelectric random access memory, i.e., into bi-stable systems comparable to the research of redox-related chemical effects. Magnetic random access memory is often based on magnetic tunnel junctions (MTJs). In MTJs, two ferromagnetic electrodes are separated by a thin insulating layer. Then, tunnel magnetoresistance can be observed, i.e., the resistance in these devices is small/large if the magnetization of two ferromagnetic layers are aligned in parallel/ antiparallel, respectively (Julliere, 1975; Moodera and Mathon, 1999). The alignment of the softer magnetic layer can be switched by current pulses because the spin of the tunneling electrons is flipped and leads to a torque, eventually switching the magnetization (Huai et al., 2004). Magnesia based MTJs exhibit on/off ratios of 2, switching voltages of approximately 500 mV and switching times in the order of ns (Kishi et al., 2008; Schaefers et al., 2009).

In the following, we try to list some of the requirements for our memristive devices. Bipolar switching is preferred, because it allows to attain STDP functionality by simple pulse shaping and overlapping (Linares-Barranco and Serrano-Gotarredona, 2009). We used the suggested pulse shaping to exemplarily demonstrate STDP using the MgO-based MTJs. The same scheme is valid for the other systems based on Ta-O. However, we increased the on-off ratio to 100% to improve the performance of the devices.

The access to a continuously varying resistance or at least multiple states would also permit the representation of synaptic weight by a single device. In either case, a large on/off ratio is desired. A write voltage of less than 3.3 V (for e.g., 350 nm technology) would be advantageous to be compatible to existing neuromorphic circuits, ideally without the requirement of a forming step. The condition that small voltages should not lead to a resistance change would be a favorable deviation from the ideal memristive behavior. Otherwise, every read process would change the resistance value of the memristive system. Finally, the memristive devices should be scalable down to nanoscopic dimensions.

If we compare the memristive tunnel junctions to these requirements, we observe bipolar switching and the access to a continuously varying resistance. The write voltages are in the order of 500 mV and no forming step is required. The junctions exhibit a voltage threshold for the resistance change and the tunnel junctions can be prepared to very small lateral dimensions. 50 × 50 nm were demonstrated already 10 years ago, and similar structures are the basis of commercially available magnetic RAM (Kubota et al., 2003). This is comparable to other technologies such as electrochemical metallization where the scalability has been demonstrated down to devices with diameters of 20 nm (Valov et al., 2011).

The amplitude of the resistance change is one obvious disadvantage of the memristive tunnel junctions if compared to the other mechanisms with on/off ratios of up to 5 orders of magnitude. Our experiments with magnesia based junctions exhibited resistance changes of less than 10%. Here, we tested new material combinations and increased the resistance change from 10% in MgO to 100% in BaTiO_3_ and Ta-O to tackle the most obvious challenge for memristive tunnel junctions as synaptic weights in future neuromorphic circuits.

## 5. Possible integration in neuromorphic systems

An ambitious aim is the full implementation of a memristive layer stack on top of a functional neuromorphic circuit. In a step toward this goal, we first contrast the different tunnel barrier materials presented in this manuscript to each other.

Magnesia was the first material where we observed memristive behavior in MTJs (Krzysteczko et al., 2008). The memristive tunnel junctions exhibited key features mimicking synaptic plasticity such as long-term depression, long-term potentiation and STDP (Krzysteczko et al., 2012; Thomas, 2013). However, the maximum amplitude between the lowest and highest resistance is 8%. This limits the use in actual devices, as discussed in the next paragraph. The main goal of the research presented in this manuscript is the preparation of memristive tunnel junctions showing larger resistance changes while maintaining the key features. Consequently, tantalum oxide and barium titanate are discussed in the following paragraphs.

Both BaTiO_3_ and Ta-O exhibit a resistance change of approximately 80% and allow access to a continuously variable property (resistance in our case), which can be used as the synaptic strength in a future device. Initially, BaTiO_3_ was chosen as one barrier material, because of the results published by Chanthbouala et al. (2012). However, the preparation process was discussed in detail, indicating a high-temperature (700°C) process. The high temperatures might complicate a full integration of the BaTiO_3_ memristors on top of existing CMOS technology, which was discussed for resistive RAM/resistive switching earlier (e.g., Pinnow and Mikolajick, 2004; Pan et al., 2014). Therefore, we focus on the Ta-O based devices.

A possible integration of memristor based devices with neuromorphic synaptic circuits was suggested by Indiveri et al. (2013). In the following, we compare the requirements pointed out in Indiveri et al. (2013) with the properties of the Ta-O based junctions. The suggested voltage is comparable to the voltage applied to our junctions. The impedance change of the memristors was assumed to be between 1 kΩ and 7 kΩ (i.e., a factor of 7) and exhibit 4 discrete resistance states, although 2 states would also be possible (Brader et al., 2007; Mitra et al., 2009). Our Ta-O devices show a resistance change of a factor of 2, which is approaching the necessary amplitudes at least for 2 resistance states. The absolute resistance of a tunnel junction is determined by the barrier thickness (with an exponential relationship) as well as the junction's area (with a linear relationship). This allows for simple tuning of the junction's resistance to the desired value by changing the junction area and barrier thickness according to the requirements of the circuit design and area constraints.

**Figure 8:**
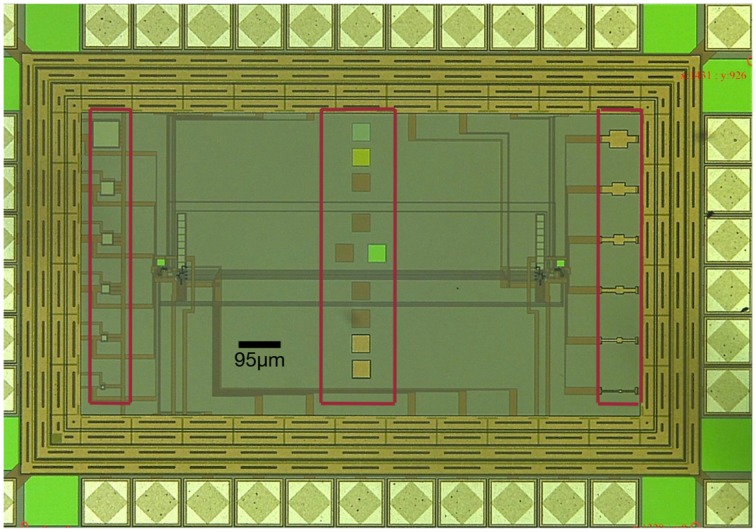
**Neuromorphic chip with contact pads for our memristors (red frames)**. The chip was fabricated using a standard AMS 0,35 μm CMOS process and supports the integration of memristor devices with neuromorphic synaptic and neural circuits.

To further demonstrate the possible integration of Ta-O memristors into existing technologies, we designed a neuromorphic chip comprised of synaptic and neural circuits as well as various test structures for the deposition of memristor devices (pads marked with red frames in Figure 8). The chip was fabricated using a standard AMS 0.35 μm CMOS process, covers an area of about 1.6 mm^2^ and includes neuromorphic circuits as described in Chicca et al. (2014). The test structures enable the deposition of the presented layer stacks on top of the chip and subsequent e-beam lithography as well as ion beam etching to define the junctions. The underlying synaptic circuits are the ones proposed by Indiveri et al. (2013) and this scheme supports the direct integration of the memristor by allowing the implementation of programmable synaptic weights. The corresponding simulation results are also given by the same authors (Indiveri et al., 2013).

Consequently, we will fully characterize the response of the synaptic and neuronal circuit for several memristor states with the goal of validating in hardware the integration, which is so far only supported by simulation results. This research will constitute an important milestone on the way toward the implementation of on chip learning algorithms capable of internally programming the synaptic weights in response to input stimuli and/or neural network dynamics.

## 6. Conclusion

In summary, we presented several materials as the functional oxide layer in tunnel junction type memristive systems. Magnesia, barium titanate and tantalum oxide indicate that these type of memristive systems can be based on many different materials. A 16 Mb magnetic random access memory is commercially available, demonstrating the good scalability of MTJs that are very similar devices.

As an example, we looked into the analogs of long-term potentiation, long-term depression and STDP in MgO based tunnel junctions. However, the maximum resistance change of up to 8% limits the applicability as synaptic weights in neuromorphic circuits. Here, we increased this resistance change by a factor of 10 in Ta-O based devices, enabling the implementation on top of a neuromorphic chip in the future.

We keep this in mind, but look into the development of autonomous neuromorphic systems now. One of the main obstacles that hindered the development of these systems is the lack of a reliable, robust, and simple implementation of a learning mechanism supported by a low-power compact device suitable for analog storage of the synaptic strength. Key requirements for learning circuits include the long time scale storage capabilities necessary to maintain acquired memories as well as mechanisms for fast modifications of synaptic weights required for the acquisition of new memories. The high integration required in large scale systems poses a severe space constraint impossible to meet with the use of digital memories and associated digital-to-analog converters.

Capacitive storage of synaptic weights has been proposed as a possible solution to this problem. A major drawback of this approach is the overhead required to compensate leakage currents affecting the charge on the capacitor. Two techniques have been proposed in this domain: reduced analog depth of the synaptic weight on long time scales (Fusi et al., 2000; Chicca et al., 2003; Giulioni et al., 2009) and switched capacitors (Vogelstein et al., 2007; Folowosele et al., 2009; Noack et al., 2015). The first solution requires redundancy and therefore large number of synapses as well as power expensive active refresh mechanisms. The second solution requires high frequency digital signals which could introduce deviations in the analog signals due to cross-talk.

Another solution that has been proposed involves the use of floating gates for synaptic strength storage (Holler et al., 1989; Diorio et al., 1998), and for the implementation of Hebbian learning (Gordon and Hasler, 2002) and STDP rules (Ramakrishnan et al., 2011; Nease et al., 2013). Floating gates synapses are suitable for large scale integration but they have the drawback of high voltages required to set the synaptic weight.

Memristors represent an emerging alternative approach to synaptic weight storage thanks to their multi-bit precision storage capability, low energy requirements for writing and nanoscale size. Therefore, we propose the integration of memristive devices in neuromorphic circuits. With this work we are laying the foundations for a possible solution to this ambitious technological challenge.

### Conflict of interest statement

The authors declare that the research was conducted in the absence of any commercial or financial relationships that could be construed as a potential conflict of interest.
